# Assessment of Human Exposure to Deoxynivalenol, Ochratoxin A, Zearalenone and Their Metabolites Biomarker in Urine Samples Using LC-ESI-qTOF

**DOI:** 10.3390/toxins13080530

**Published:** 2021-07-28

**Authors:** Dionisia Carballo, Noelia Pallarés, Emilia Ferrer, Francisco J. Barba, Houda Berrada

**Affiliations:** 1 Faculty of Agricultural Science, National University of Asunción, San Lorenzo 2160, Paraguay; dionisia.carballo@agr.una.py; 2 Department of Preventive Medicine and Public Health, Food Science, Toxicology and Forensic Medicine, Faculty of Pharmacy, University of Valencia, 46100 Burjassot, Valencia, Spain; Noelia.pallares@uv.es (N.P.); houda.berrada@uv.es (H.B.)

**Keywords:** mycotoxins, biomarkers, metabolites, LC-ESI-qTOF, urine, risk assessment

## Abstract

Human are exposed to a wide range of mycotoxins through dietary food intake, including processed food. Even most of the mycotoxin exposure assessment studies are based on analysis of foodstuffs, and evaluation of dietary intake through food consumption patterns and human biomonitoring methods are rising as a reliable alternative to approach the individual exposures, overcoming the limitations of the indirect dietary assessment. In this study, human urine samples were analyzed, seeking the presence of deoxynivalenol (DON), ochratoxin A (OTA), zearalenone (ZEA), and their metabolites. For this purpose, 40 urine samples from female and male adult residents in the city of Valencia (Spain) were evaluated by liquid chromatography quadrupole time-of-flight mass spectrometry (LC-ESI-qTOF) after salting-out liquid–liquid extraction. Analytical data showed that 72.5% of analyzed samples were contaminated by at least one mycotoxin at variable levels. The most prevalent mycotoxins were de-epoxy DON (DOM-1) (53%), ZEA (40%), and α-zearalenol (αZOL) (43%), while OTA was only detected in one sample. The mean concentrations in positive samples were DON (9.07 ng/mL), DOM-1 (20.28 ng/mL), ZEA (6.70 ng/mL), ZEA-14 glucoside (ZEA-14-Glc) (12.43 ng/mL), αZOL (27.44 ng/mL), αZOL-14 glucoside (αZOL-14-Glc) (12.84 ng/mL), and OTA (11.73 ng/mL). Finally, probable daily intakes (PDIs) were calculated and compared with the established tolerable daily intakes (TDIs) to estimate the potential risk of exposure to the studied mycotoxins. The calculated PDI was below the TDI value established for DON in both female and male adults, reaching a percentage up to 30%; however, this percentage increased up to 92% considering total DON (DON + DOM-1). On the other hand, the PDI obtained for ZEA and its metabolites were higher than the TDI value fixed, but the low urine excretion rate (10%) considered should be highlighted. Finally, the PDI calculated in the detected positive sample for OTA exceeded the TDI value. The findings of the present study confirm the presence of the studied mycotoxins and their metabolites as some of the most prevalent in urine.

## 1. Introduction

Mycotoxins are toxic secondary metabolites produced by various fungi on diverse agricultural commodities. The accumulation of mycotoxins in food and feedstuffs represent a major threat to human and animal health, as they are related to different toxic effects, such as cancer induction, mutagenicity, nephrotoxicity, estrogenicity, and urogenital and nervous disorders. Contaminated food consumption is the major source of human exposure to mycotoxins [1,2]. Thus, the food industry is looking for technologies that can remove mycotoxins from food materials [3].

Traditional exposure assessment to mycotoxins involves foodstuff analysis and the estimation of average consumption patterns. However, this approach presents some disadvantages, such as the lack of information at individual exposure, and the bioavailability and toxicokinetics [4]. An alternative way to evaluate the exposure can be through biomarkers. A biomarker exposure is defined as a biological measure that is correlated with the quantity of toxic compound ingested [5]. Biomarkers allow more accurate assessment of the exposure at an individual level because their measurements include the individual variation in absorption, distribution, metabolism, and excretion [6,7].

Typical biomarkers include parent toxins themselves, protein or DNA adducts, and/or the major phase I or II metabolites (glucuronide conjugates) that may be measured in biological fluids, such as plasma/serum or urine, and are related to the intake of mycotoxins through contaminated food [4]. In this context, the analysis of mycotoxins and their metabolites in human urine may provide a very suitable alternative for the evaluation of exposure to mycotoxins. In addition, urine collection constitutes an easy and noninvasive sampling method.

Biomarkers of mainly reported mycotoxins have been previously studied in urine [8,9,10,11]. In fact, deoxynivalenol (DON), zearalenone (ZEA), ohratoxin A (OTA), and their selected metabolites are some of the most frequently reported mycotoxins in urine. ZEA commonly occurs in various cereal crops and processed grains (i.e., wheat, rice, and corn). It produces estrogenic effects, which can have adverse effects on the genital organs and reproductive system. After oral administration, ZEA is rapidly absorbed and subsequently degraded into α-zearalenol (α-ZOL) and β-zearalenol (β-ZOL), which may be additionally reduced into α-zearalanol (α-ZAL) and β-zearalanol (β-ZAL). α-ZAL is metabolized into its isomer, β-ZAL, and, to a lesser extent, into zearalanone (ZAN) [12]. These compounds are partially conjugated with sulfonic or glucuronic acid to produce, i.e., ZEA-14-glucuronic acid (ZEA-14-GlcA), ZAN-14-GlcA, α-ZOL-14- GlcA, β-ZOL-14- GlcA, that are excreted in the urine [13]. The human urinary excretion of ZEA corresponds to approximately 10% of the administrated dose, ZEA glucuronides and α-ZOL being the main metabolites excreted [14].

DON is a natural contaminant of cereal grains (i.e., maize, wheat, barley, and oats) and cereal-based processed food (i.e., cereals, bread, and mat beer). DON is related to adverse effects, including vomiting, nausea, anorexia, delayed growth, neurological changes, and impairments in reproductive and immune function. DON has a short life of excretion; it is detectable in high amounts in the serum immediately after ingestion, but it is rapidly cleared from the bloodstream. The main fraction of DON can be excreted in urine in its unmetabolized form, but also as glucuronide conjugates (DON-GlcA) including the deoxynivalenol-3-glucuronide (DON-3-GlcA) and deoxynivalenol-15-glucuronide (DON-15-GlcA). Furthermore, the de-epoxy DON (DOM-1), a detoxification product of DON formed by gut microbiota, can also be detected in urine [15]. In humans, the estimated percentage of excretion corresponds to 72.3% of the administered dose [16].

OTA is a natural contaminant of many foodstuffs, such as cereal products, coffee, cacao, beer, grape juice, raisins, wine, and spices. Regarding its toxicity, OTA has been associated with nephrotoxic and carcinogenic adverse effects. OTA is biotransformed by cytochrome P450 enzymes to their hydroxyochratoxin A metabolites, which are less toxic. In humans, ochratoxin alpha (OTα) is the major metabolite of OTA and is formed in the gastrointestinal tract by hydrolysis. OTα is further metabolized to a glucuronide and possibly to a sulfate conjugate, being more efficiently cleared from the circulation system and easily excreted in the urine [17]. The percentage of OTA excreted in urine is nearly 50% of the OTA ingested [18].

Enzymatic hydrolysis with β-glucuronidase is required to release the parent mycotoxin from the mycotoxins–glucuronides. After enzymatic deconjugation, the total amount of (free+conjugated) mycotoxins and their metabolites can be used to assess mycotoxin exposure.

This study was conducted with the aim of assessing human exposure to mycotoxins through multi-biomarker analysis in 40 urine samples of different volunteers. For this purpose, urine samples were extracted by salting-out liquid–liquid extraction (SALLE) and determined using liquid chromatography coupled with quadrupole time-of-flight mass spectrometry with electrospray ionization (LC-ESI-QTOF-MS) system. Finally, to estimate the potential risk of exposure to the studied mycotoxins, the probable daily intakes (PDIs) were calculated and compared with the tolerable daily intakes (TDIs) established.

## 2. Results and Discussion

### 2.1. Mycotoxin Contents in Urine

**Figure 1:**
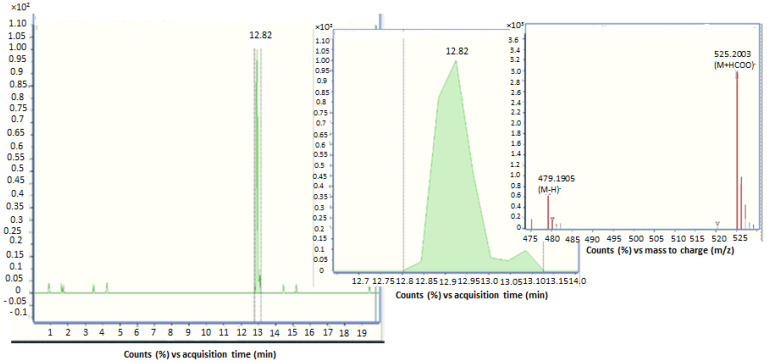
LC-ESI-qTOF-MS chromatogram of a human urine sample naturally contaminated by zearalenone-14-glucoside (ZEA-14-Glc).

DON, DOM-1, ZEA, zearalenone-14-glucoside (ZEA-14-Glc), αZOL, α-zearalenol-14-glucoside (αZOL-14-Glc), and OTA were the main mycotoxins and metabolites identified in the urinary samples. The incidence, minimum and maximum concentrations, and mean of positives are shown in Table 1. Among all, ZEA and its metabolites ZEA-14-Glc, αZOL, αZOL-14-Glc, and DOM-1 were the biomarkers with the highest incidence in urinary samples, showing percentages of 40%, 18%, 43%, 20%, and 53%, respectively. These biomarkers were detected in urinary samples with concentrations between <LOQ and 49.45 ng/mL. Figure 1 shows the chromatogram of a human urine sample containing ZEA-14-Glc.

Regarding ZEA and its metabolites, the mean concentration of positives determined in the present work were 6.7 ng/mL for ZEA, 12.43 ng/mL for ZEA-14-Glc, 27.44 ng/mL for αZOL, and 12.84 ng/mL for αZOL-14-Glc (Table 1). These values were higher than those observed by Li et al. [12], even though they did not include hydrolyzed samples. These authors reported very low incidences for ZEA and αZOL (1.3 %), with amounts ranging from <LOQ to 0.05 ng/mL. Moreover, they detected ZAN (0.3%) and βZOL (1%), but these two ZEA metabolites were not identified in the present study. In another study, performed by Solfrizzo et al. [19] in hydrolyzed samples, higher incidences (about 100%) were found for βZOL, αZOL, and ZEA, but at lower mean concentrations, with values of 0.090, 0.077, and 0.057 ng/mL, respectively. Ediage et al. [8] also reported lower incidences (around 10%) but more similar values than those obtained in the present study, despite the fact that ZEA-14-Glc, αZOL, and αZOL-14-Glc were not detected by these authors. ZEA and βZOL were reported at contents ranging from <LOQ to 12.6 ng/mL and from 4 to 24.8 ng/mL, respectively, by these authors. Sarkanj et al. [20] observed similar contents to the present study and a higher incidence for ZEA in hydrolyzed urine samples, with contents ranging from 0.03 to 19.99 ng/mL and an incidence of 81.7%; however, lower incidences and contents were reported for αZOL, at 4.2 % and up to 2.52 ng/mL.

OTA was only detected in one sample, at a concentration of 11.73 ng/mL (Table 1). Comparing these results with the information available in the literature, the contents reported in the available literature are, in general, lower. Ali et al. [17] reported all positive samples for OTA in the range of 0.02–1.82 ng/mL. However, higher amounts were observed for OTα (0.01–14.25 ng/mL). Other studies have also reported notable incidence for OTA, even at lower levels, below 0.14 ng/mL [10,19].

DOM-1, the metabolite produced from DON by intestinal microbiota, was detected in 21 of 40 analyzed samples (53%), with a positive mean of 20.28 ng/mL (Table 1). In another study, performed in Spain, Vidal et al. [10] registered DOM-1 with high incidences, at 96% of urine samples of volunteers following a normal diet, and at 78% of urine samples of volunteers with a temporary diet restriction, including food commonly associated with mycotoxins presence. The mean concentrations determined were 23 and 12.9 ng/mL, respectively. In France, Turner et al. [21] also obtained a lower percentage (34%) of positive urine samples, with a mean concentration of 0.2 ng/mL. In the UK, Turner et al. [22] only observed 2% of urinary samples as positive, at a mean concentration of 0.65 ng/mL. Contrary to the present study, Cunha et al. [23] did not find DOM-1 in any of the analyzed urinary sample.

The parent mycotoxin, DON, was detected in the present work in 9 of 40 analyzed samples (23%), with a mean of positives of 9.07 ng/mL (Table 1). Similar results were previously reported by Rodriguez-Carrasco et al. [24], who quantified a mean of positive samples for free DON of 7.9 ng/mL in 50% of analyzed urinary samples. Moreover, Solfrizzo et al. [19] observed that 96% of samples were positive for DON after the hydrolysis process, with a mean concentration of 11.89 ng/mL. Contrary to these results, Warth et al. [25] did not determine free DON in analyzed urinary samples. Niknejad et al. [26] studied the multi-mycotoxin urinary levels in healthy volunteers and esophageal cancer patients, and reported DON presence in only one sample from healthy volunteers at a concentration of 8.42 µg/L.

### 2.2. Exposure Assessment

The calculated PDI values for ZEA and total ZEA (ZEA and its metabolites: ZEA-14-Glc, αZOL, αZOL-14-Glc) in positive samples were, for both males and females, higher than the TDI established (0.25 µg/kg bw/day). This was also observed when the PDI was calculated under LB and UB scenarios (Table 2). However, the low ZEA excretion percentage in urine (10%) considered to calculate the PDI should be highlighted. Contrary to these results, in Italy, Solfrizzo et al. [19] estimated a mean PDI value in urine samples about ten times below the TDI established; however, these authors considered a higher mean urinary excretion rate for ZEA (36.8%).

Regarding OTA, only one sample was positive, but the PDI estimated was 0.42 µg/kg bw/day for males and 0.52 µg/kg bw/day for females, exceeding the tolerable weekly intake (TWI) of 0.12 µg/kg bw/week; however, 8.3% and 10.8% of TDI was reached for males and females, respectively, when PDI was calculated under the LB scenario, and 50% and 66.7% in the UB (Table 2). Similar to these results, Solfrizzo et al. [19] observed that the TDI value calculated for OTA in urine exceeded the TWI fixed in 94% of volunteers. Contrary to these results, Heyndrickx et al. [6] observed that only 1% of Belgian volunteers’ urine exceeded the TWI for OTA.

Finally, the PDI calculated for DON was a mean of 0.25 µg/kg bw/day for males and 0.30 µg/kg bw/day for females, reaching a percentage of up to 30% of the TDI fixed for DON. All DON metabolites were included in the assessment of the total DON amount (DON + DOM-1). Thus, PDI values increased to 0.76 and 0.92 µg/kg bw/day for males and females, respectively, considering total DON, representing up to 92% of the TDI. These percentages of TDI decreased to up to 6.7% (DON) and 40.2% (total DON) when the PDI was calculated considering the LB and UB scenarios (Table 2).

A similar range was also observed by Warth et al. [24], who reported exposure percentages of TDI for total DON (free DON + DON-GlcAs) in the range of 38% to 220% in a study carried out in Austria. In a previous study, Rodríguez-Carrasco et al. [27] obtained similar results. These authors calculated the DON PDI as DON + DOM-1 levels and observed that these represented a range of between 6% and 107% of the established TDI. Furthermore, 8.1% of the total exposed subjects exceed the TDI limit. In Germany, Gerding et al. [28] observed that 12% of the urine samples exceeded the TDI value fixed for DON, considering DON-GlcA and DON biomarkers. Contrary to these results, Fan et al. [29] obtained a mean PDI value of 0.164 μg/kg bw/day for DON in a study performed in China, observing that only 0.4% of the population exceeded the TDI value.

Comparing these results with total diet studies, in Catalonia (a region of Spain), where mycotoxin dietary intake was assessed in order to characterize mycotoxin exposure under a deterministic methodology through the combination of data consumption with contamination levels, among all mycotoxins, OTA and DON constitute the most significant, reaching TDI percentages from 1 to 17% for OTA and up to 74% for DON [30]. These mycotoxins have also been reported in the present study as important biomarkers. However, ZEA estimated daily intakes (EDIs) reported by these authors were far below the TDI established. The approach proposed in the present study overcomes some disadvantages, such as the lack of information related to the individual exposure situation, toxicokinetics, and bioavailability. Moreover, mycotoxin distribution in food is not homogeneous and it is complicated to obtain accurate data on food consumption [31].

## 3. Materials and Methods

### 3.1. Chemicals and Reagents

Acetonitrile (AcN) and methanol (MeOH) were supplied by Merck (Darmstadt, Germany). Deionized water (<18.2 MΩ cm resistivity) was obtained in the laboratory using a Milli-QSP^®^ Reagent Water System (Millipore, Beadford, MA, USA). Formic acid (reagent grade ≥95%) was obtained from Sigma-Aldrich (St. Louis, MO, USA). Sodium chloride and ammonium acetate sorbent (analytical grade) were obtained from Merck. C18 was purchased from Phenomenex (Torrence, CA, USA). β-Glucuronidase Type H-1 from Helix pomatia (glucuronidase activity: ≥300,000 units/g solid and sulfatase activity: ≥10,000 units/g solid) was supplied by Sigma Aldrich. Syringe nylon filters (13 mm diameter 0.22 µm pore size) were obtained from Analysis Vinicos S.L. (Tomelloso, Spain).

### 3.2. Standards and Solutions

The powder standards of ZEA, DON, DOM-1, and OTA were purchased from Sigma Aldrich and reconstituted in methanol at a concentration of 100 mg/L. Then, individual stock of all analytes were prepared to obtain 20 mg/L in methanol and multianalyte working solutions of 2 mg/L were also used by diluting the individual stock solutions in methanol. The multianalyte working standard solution was used for standard calibration curves, matrix-matched calibration curves, and recovery assays. All standards were stored in darkness and kept at −20 °C.

### 3.3. Sampling

Forty first-spot morning urine samples were collected during February and March 2019 from the Spanish population resident in Valencia region. Samples were acquired from a group of 20 males and 20 females within the age range of 18–65. All urine samples were collected into sterile plastic vessels. The samples were stored in a freezer (−20 °C) until analysis. The volunteers did not consume any special diet on the day prior to sample donation. All samples were anonymous, but participants indicated their age, weight, and gender, male (M) or female (F). A written and approved informed consent was obtained from the volunteers. 

### 3.4. Sample Preparation

#### 3.4.1. Samples Enzymatic Hydrolysis

All urine samples were centrifuged at 10,000 rpm for 3 min at 4 °C.

Samples were hydrolyzed according to a previous study [24]. For this, 1 mL of the previously centrifuged urine was collected in a 2 mL Eppendorf tube and 250 µL of ammonium acetate buffer (1 M, pH5) containing 20,000 U of β-glucuronidase/mL was added. The hydrolysis was performed with continuous stirring at 550 rpm during 18 h at 37 °C.

#### 3.4.2. Salting-Out Assisted Liquid–Liquid Extraction (SALLE)

After enzymatic hydrolysis, the sample was centrifugated and the upper layer was placed into a 15 mL conical bottom tube. Then, 1 mL of acetonitrile and a mixture of 0.3 g sodium chloride and 30 mg of C18 sorbent were added. The mixture was vortexed for 30 s and centrifugated at 4000 rpm for 3 min at 4 °C. Finally, the upper layer was evaporated to near dryness under a nitrogen stream using a TurboVap LV Evaporator (Zimark, Hopkinton, MA, USA). The dry residue was reconstituted with 0.5 mL of MeOH/H2O (50/50 *v*/*v*) and filtrated through a 13-mm/0.22-μm nylon filter prior to LC-ESI-qTOF-MS analysis.

### 3.5. LC-ESI-qTOF-MS Analysis

An Agilent 1200-LC system (Agilent Technologies, Palo Alto, CA, USA) equipped with a vacuum degasser, autosampler, and binary pump was used for the chromatographic determination. The column was Gemini^®^ NX-C18 (3 µM, 150 × 2 mm ID) and guard column C18 (4 × 2 mm, ID; 3 µM) (Phenomenex). The mobile phases consisted of water (A) and acetonitrile (B), with 0.1% in formic acid for both. The gradient program was as follows: 0–6 min, 50% B; 7–12 min, 100% B; 13–20 min, 50% B. The injection volume for standards and sample extracts was 5 µL and the flow rate used was 0.2 mL/min. A mass spectrometry (MS) analysis was carried out using a 6540 Agilent Ultra-High-Definition Accurate-Mass q-TOF-MS coupled to the HPLC, equipped with an Agilent Dual Jet Stream electrospray ionization (Dual AJS ESI) interface in positive and negative ionization modes under the following conditions: drying gas flow (N2), 12.0 L min^−1^; nebulizer pressure, 50 psi; gas drying temperature, 370 °C; capillary voltage, 3500 V; fragmentor voltage, 160 V; and scan range *m*/*z*, 50–1500. Automatic MS/MS experiments were carried out using the following collision energy values: *m*/*z* 100, 30 eV; *m*/*z* 500, 35 eV; *m*/*z* 1000, 40 eV; and *m*/*z* 1500, 45 eV. Integration and data elaboration were performed using Mass Hunter Workstation software (Agilent Technologies). The analytical and spectrometric parameters are listed in Table 3.

MassHunter Qualitative Analysis program was employed to identify the mycotoxins and their metabolites in the urine samples, employing an accurate mycotoxin mass library, considering only the matching compounds identified with a minimum score of 80. Then, matrix-matched calibration curves constructed at concentrations of the parent compound ranging from LOD to 1000 µg/L were used for effective quantification of samples.

### 3.6. Risk Assessment Using Mycotoxins Biomarker Quantification

The probable daily intakes (PDIs) of mycotoxins among the participants were obtained based on results of mycotoxin biomarkers in urine, employing the following equation according to Solfrizzo et al. [19]:

PDI (µg kg bw/day) = C × V × 100/W × E(1)

where C is the concentration of the mycotoxin biomarker in urine (µg/L), V is the mean volume of urine excreted in 24 h, established in 1.5 L according to Rodríguez-Carrasco et al. [27]. W is the body weight (kg), considering a mean of 67.2 kg for females and 82 kg for males based on the EFSA guidance [32]. E refers to the excretion rate (%) of the corresponding mycotoxins, calculated as 10% approximately for ZEA [14], around 72.3% for DON [16], and as 50% for OTA [18]. Variation in excretion rates, as well as urine flow rate excreted among individuals, have not been considered to calculate PDIs.

Two more scenarios were considered, lower bound scenario (LB) and upper bound scenario (UB) to process data below LOD according to EFSA recommendations [33]. In the LB scenario, zero was assigned when mycotoxins were not detected or were detected below the limit of quantification, while the limit of detection was assigned in the UB scenario.

Then, the calculated probable daily intakes (PDIs) were compared to the tolerable daily intakes fixed by the European Commission for the studied mycotoxins to assess the risk of exposure. A TDI of 0.25 µg/kg bw/day was established for ZEA [34] and of 1 µg/kg bw/day for DON [35], while a tolerable weekly intake (TWI) of 0.12 µg/kg bw/week has been fixed for OTA [36].

## 4. Conclusions

The analytical method based on salting-out liquid–liquid extraction and LC-ESI-qTOF allowed the detection of DON, ZEA, OTA, and their metabolites as DOM-1, ZEA-14-Glc, αZOL, and αZOL-14-Glc in urine samples. The incidence of these biomarkers ranged from 3% for OTA to 53% for DOM-1, while the concentrations were comprised between ˂LOQ and 49.45 ng/m. The calculated PDI values for OTA and ZEA and their metabolites were, in some urine samples, greater than the established TDI values. These results confirm that these mycotoxins and their metabolites are still prevalent in urine and the development of biomarker approaches will greatly help in evaluating human exposure to mycotoxins, among other food contaminants.

## Figures and Tables

**Table 1:** Incidence (%), minimum and maximum levels (ng/mL), and mean of positives (ng/mL) of mycotoxins and its metabolites detected in urinary samples.

Mycotoxin	Number and Percentage of Positive Samples	Range Concentration (ng/mL)	Mean of Positives (ng/mL)
DON	9/40 (23%)	<LOQ–18.67	9.07 ± 7
DOM-1	21/40 (53%)	<LOQ–49.45	20.28 ± 4
ZEA	16/40 (40%)	<LOQ–29.01	6.70 ± 7
ZEA-14-Glc	7/40 (18%)	2.87–28.80	12.43 ± 9
αZOL	17/40 (43%)	<LOQ–43.68	27.44 ± 20
αZOL-14-Glc	8/40 (20%)	<LOQ–29.20	12.84 ± 14
OTA	1/40 (3%)	11.73	11.73

**Table 2:** Mycotoxins risk assessment based on the mycotoxin biomarker urinary levels among the participants.

		Mean Positive Samples				Lower Bound Scenario				Upper Bound Scenario			
Mycotoxin	Tolerable Daily Intake (µg/ kg bw/day)	Mean PDI (µg/kg bw/day		%TDI		Mean PDI (µg/kg bw/day		%TDI		Mean PDI (µg/kg bw/day		%TDI	
		Males	Females	Males	Females	Males	Females	Males	Females	Males	Females	Males	Females
DON	1	0.25	0.30	25%	30%	0.052	0.063	5.2%	6.3%	0.055	0.067	5.5%	6.7%
Total DON: (DON+DOM-1)	1	0.76	0.92	76%	92%	0.322	0.393	32.2%	39.3%	0.329	0.402	32.9%	40.2%
ZEA	0.25	1.23	1.50	492%	600%	0.49	0.6	196%	240%	0.53	0.64	212%	256%
Total ZEA: (ZEA, ZEA-14 -Glc, αZOL, αZOL-14 -Glc)	0.25	10.87	13.27	4348%	5308%	3.49	4.25	1396%	1700%	3.66	4.46	1464%	1784%
OTA	0.12	0.42	0.52	350%	433%	0.01	0.01	8.3%	10.8%	0.06	0.08	50%	66.7%

**Table 3:** Retention times, molecular formula, measured neutral mass, observed mass in ionization mode, mass accuracy, and detection and quantification limits.

Mycotoxin	Retention Time (min)	Molecular Formula	Measured Neutral Mass (*m*/*z*)	Observed Mass in Ionization Mode		Accuracy (Δ ppm)	LOD ng/mL	LOQ ng/mL
OTA	7.3	C_20_H_18_ClNO_6_	403.0839	(M-H)^−^	402.0768	2.3	1.5	5
ZEA	7.9	C_18_H_22_O_5_	318.1467	(M-H)^−^	317.1394	1.03	0.33	1
ZEA-14-Glc	12.9	C_24_H_32_O_10_	480.1941	(M-H)^−^	479.1922	4.6	-	-
αZOL	4.68	C_18_H_24_O_5_	320.1623	(M-H)^−^	319.1551	−2.6	0.33	1
αZOL-14-Glc	12.93	C_24_H_34_O_10_	482.2151	(M-H)^−^	481.2082	2.86	-	-
DON	1.738	C_15_H_20_O_6_	296.1277	(M+HCOOH)^−^	341.1258	−2.5	0.15	0.5
DOM-1	2.09	C_15_H_20_O_5_	280.1303	(M+H)^+^	281.1376	2.5	0.33	1
